# The impact of tumor size on the survival of patients with small renal masses: A population–based study

**DOI:** 10.1002/cam4.4595

**Published:** 2022-03-01

**Authors:** Yiming Tang, Fei Liu, Xiaopeng Mao, Pengju Li, Mukhtar A. Mumin, Jiaying Li, Yi Hou, Hongde Song, Haishan Lin, Lei Tan, Chengpeng Gui, Mingxiao Zhang, Liangmin Fu, Wei Chen, Yong Huang, Junhang Luo

**Affiliations:** ^1^ Department of Urology, The Second Affiliated Hospital Guangzhou Medical University Guangzhou China; ^2^ Department of Urology, The First Affiliated Hospital Sun Yat‐sen University Guangzhou China; ^3^ Department of Urology, National Cancer Center/National Clinical Research Center for Cancer/Cancer Hospital Chinese Academy of Medical Sciences and Peking Union Medical College Beijing China; ^4^ Department of Emergency, The First Affiliated Hospital Sun Yat‐sen University Guangzhou China

**Keywords:** intervention, outcome, SEER, small renal masses, tumor size

## Abstract

**Background:**

Active surveillance (AS) with delayed intervention has gained acceptance as a management strategy for small renal masses (SRMs). However, during AS, there is a risk of tumor growth. Thus, we aim to investigate whether tumor growth in patients with SRMs leads to tumor progress.

**Methods:**

In this study, we enrolled 16,070 patients from the Surveillance, Epidemiology, and End Results database with T1a renal cell carcinoma (RCC) between 2004 and 2017. The 16,070 patients were divided into three groups: 10,526 in the partial nephrectomy (PN) group, 2768 in the local ablation (LA) group, and 2776 in the AS group. Associations of tumor size with all‐cause and cancer‐specific mortality were evaluated using Kaplan–Meier analyses and Cox regression models.

**Results:**

Four tumor size categories were delineated (≤1, >1–2, >2–3, and > 3–4 cm in diameter), and 10‐year all‐cause and cancer‐specific mortality both significantly increased with increasing tumor size in the PN, LA, and AS groups (all *p* < 0.05). Tumors were substaged based on diameter: T1aA (≤2 cm) and T1aB (>2–4 cm). All‐cause and cancer‐specific mortality were significantly higher in T1aB tumors than T1aA tumors in each group (hazard ratio = 1.395 and 1.538, respectively; all *p* < 0.05).

**Conclusions:**

Tumor growth relates to worse prognosis of T1a RCC, and 2 cm serves as a size threshold that is prognostically relevant for patients with T1a RCC. Because of the lack of accurate predictors of tumor growth rate, AS for patients with SRMs incurs a risk of tumor progression.

## Graphical abstract

Our research used a large‐sample dataset (including 16,070 patients) to reconstruct a new sub‐stage system of Stage T1a RCC with the cut‐off of 2 cm, which may accurately indicate tumor progression and provide a basis for the treatment of small renal masses.

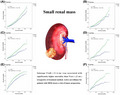

## 1 INTRODUCTION

Renal cell carcinoma (RCC) is the most common malignant tumor of the adult kidney, accounting for 2% to 3% of all cases, and remains one of the most lethal malignancies of urinary system tumors.^1^ A solid‐enhancing tumor with a maximum axial diameter of ≤4 cm, which is usually consistent with stage T1a RCC, is defined as a small renal mass (SRM).^2^ In the past 20 years, the surgical treatment of SRMs has changed from radical nephrectomy (RN) to partial nephrectomy (PN) or local ablation (LA), if technically feasible.^3^

Over the years, active surveillance (AS) has been increasingly recommended as an initial treatment option for patients with SRMs, especially for patients with tumors smaller than 2 cm in diameter.^4^, ^5^ Several studies suggested that not all SRMs require primary intervention (PI) because the incidence of metastasis and mortality was similar between PI and AS with delayed intervention (DI), even though AS has a risk of tumor growth.^6^, ^7^, ^8^, ^9^ However, the average tumor size of the AS group was significantly smaller than that of the PI group in these studies (1.5 cm vs. 2.5 cm; *p* < 0.01 and 1.9 cm vs. 2.5 cm; *p* < 0.05).^6^, ^9^ These results might reflect the comfort of patients and urologists with AS as a management strategy, but the value of these findings is probably limited by the inevitable selection bias. Furthermore, owing to the small sample sizes in these studies, the findings regarding effects of AS and DI might be unconvincing.^6^, ^7^, ^8^ Moreover, tumor size was not static during AS, and other studies reported that RCC grows at a mean rate of 0.09–0.86 cm per year.^10^, ^11^, ^12^ Different outcomes of AS might result because of the different tumor growth speeds of individual masses, and there is no accurate predictor of tumor growth speed during AS. Therefore, whether AS actually benefit SRM patients is yet to be fully determined due to the uncertainty of the tumor size.

In this study, we investigated 16,070 SRMs in the Surveillance, Epidemiology, and End Results (SEER) cancer database to determine whether tumor growth in patients with T1a RCC led to tumor progression. In view of the individual differences in demographic and clinical characteristics and biological characteristics of SRM patients, we also conducted subgroup analysis and prognostic risk assessment of patients in different tumor size.

## 2 PATIENTS AND METHODS

This study was based on the SEER cancer database, sponsored by the U.S. National Cancer Institute (https://seer.cancer.gov/). Because SEER data are anonymous, this study did not require institutional review board approval. All data were downloaded using SEER*Stat software (version 8.3.8) under accession number 14845‐Nov2019.

Using the SEER database, we identified patients diagnosed with stage T1a RCC (measuring ≤4 cm in diameter) from 2004 to 2017. The data search was limited to patients who underwent PN, LA, or AS. Patients were excluded from the analysis for any of the following reasons: (1) having tumor extension outside the kidney or any nodal or distant metastases; (2) lacking detailed information on tumor size; (3) having a diagnosis that was made only at the time of death; or (4) lacking follow‐up information. The factors included from the SEER database were age, gender, grade, tumor size, histological cell type, and treatment method.

The primary and secondary outcomes were all‐cause mortality and cancer‐specific mortality, respectively. Cause of death was determined based on the list of cases in the SEER database and was classified as attributable to cancer or secondary to another cause. All‐cause mortality was defined as all deaths that occur in a population, irrespective of the cause; cancer‐specific mortality was defined as all deaths that occur in a population from RCC. Mean (±SD) and frequency (%) were used to describe continuous and categorical variables, respectively. Student's *t*‐test was used to compare continuous variables between two groups, depending on whether the continuous variable data were normally distributed. Categorical variables were compared with the chi‐squared test. The Kaplan–Meier (KM) method using log‐rank statistics was used to compare all‐cause mortality and cancer‐specific mortality among different tumor sizes in the PN, LA, and AS groups. Cox proportional hazard models were used to estimate hazard ratios (HRs). HRs and 95% confidence intervals (CIs) were reported. All analyses were conducted using SPSS version 22.0 software (IBM, SPSS Statistics). All *p* values are two sided, and *p* < 0.05 was used as the threshold for statistical significance.

## 3 RESULTS

**FIGURE 1:**
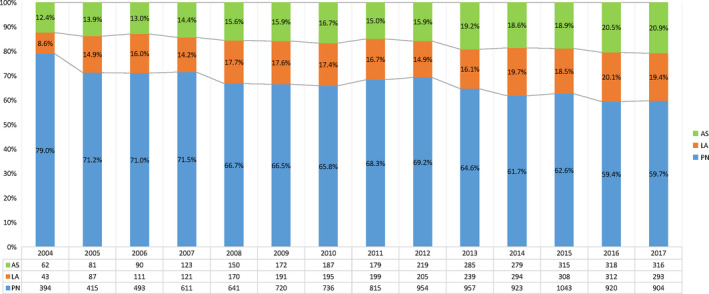
Proportion of treatment with the year at diagnosis in patients with SRMs. Stacked bar chart shows that the proportion of AS increased with the year of diagnosis, from 12.4% in 2004 to 20.9% in 2017. The proportion of PN decreased from 79.0% in 2004 to 59.7% in 2017

We examined 16,070 patients who underwent PN (10,526), LA (2768), or AS (2776) for T1aN0M0 RCC from 2004 to 2017. The clinical features of patients included age, gender, grade, histological subtype, and tumor size, which are summarized in Table 1. AS and LA were used more often for older patients—for 82.2% and 77.2% of patients over 60 years of age, respectively—whereas patients who underwent PN tended to be younger, accounting for 52.1% of patients under 60 years of age. Median follow‐up for the whole cohort was 51 months (interquartile range: 23–88 months; range: 1–167 months). The proportion of AS increased with the year of diagnosis, from 12.4% in 2004 to 20.9% in 2017 (Figure 1). Especially in those tumors smaller than 2 cm in diameter, the selection rate of AS was increased more obviously (24.2% in 2017 vs. 19.4% in 2017) than for tumors larger than 2 cm in diameter (Figures S1 and S2).

**TABLE 1:** Clinical and pathological features of patients with T1a RCC

Variables	Treatment		
	PN (10,526)	LA (2768)	AS (2776)
Age, years			
<60	5487 (52.1%)	632 (22.8%)	493 (17.8%)
≥60	5039 (47.9%)	2136 (77.2%)	2283 (82.2%)
Gender			
Female	3968 (37.7%)	1036 (37.4%)	1026 (37.0%)
Male	6558 (62.3%)	1732 (62.6%)	1750 (63.0%)
Grade			
I	1698 (16.1%)	457 (16.5%)	149 (5.4%)
II	5615 (53.3%)	848 (30.6%)	272 (9.8%)
III	1820 (17.3%)	133 (4.8%)	46 (1.7%)
IV	98 (0.9%)	8 (0.3%)	5 (0.2%)
Unknown	1295 (12.3%)	1322 (47.8%)	2304 (83.0%)
Histological subtypes			
Clear cell	6457 (61.3%)	1297 (46.9%)	492 (17.7%)
Papillary	1866 (17.7%)	490 (17.7%)	196 (7.1%)
Chromophobe	682 (6.5%)	88 (3.2%)	47 (1.7%)
RCC undefined	1180 (11.2%)	791 (28.6%)	1869 (67.3%)
Other histologies	341 (3.2%)	102 (3.7%)	172 (6.2%)
Tumor size, cm			
≤1	371 (3.5%)	44 (1.6%)	95 (3.4%)
>1–2	3388 (32.2%)	834 (30.1%)	885 (31.9%)
>2–3	4179 (39.7%)	1263 (45.6%)	1113 (40.1%)
>3–4	2588 (24.6%)	627 (22.7%)	683 (24.6%)

Abbreviations: AS, active surveillance; LA, local ablation; PN, partial nephrectomy; RCC, renal cell carcinoma.

**FIGURE 2:**
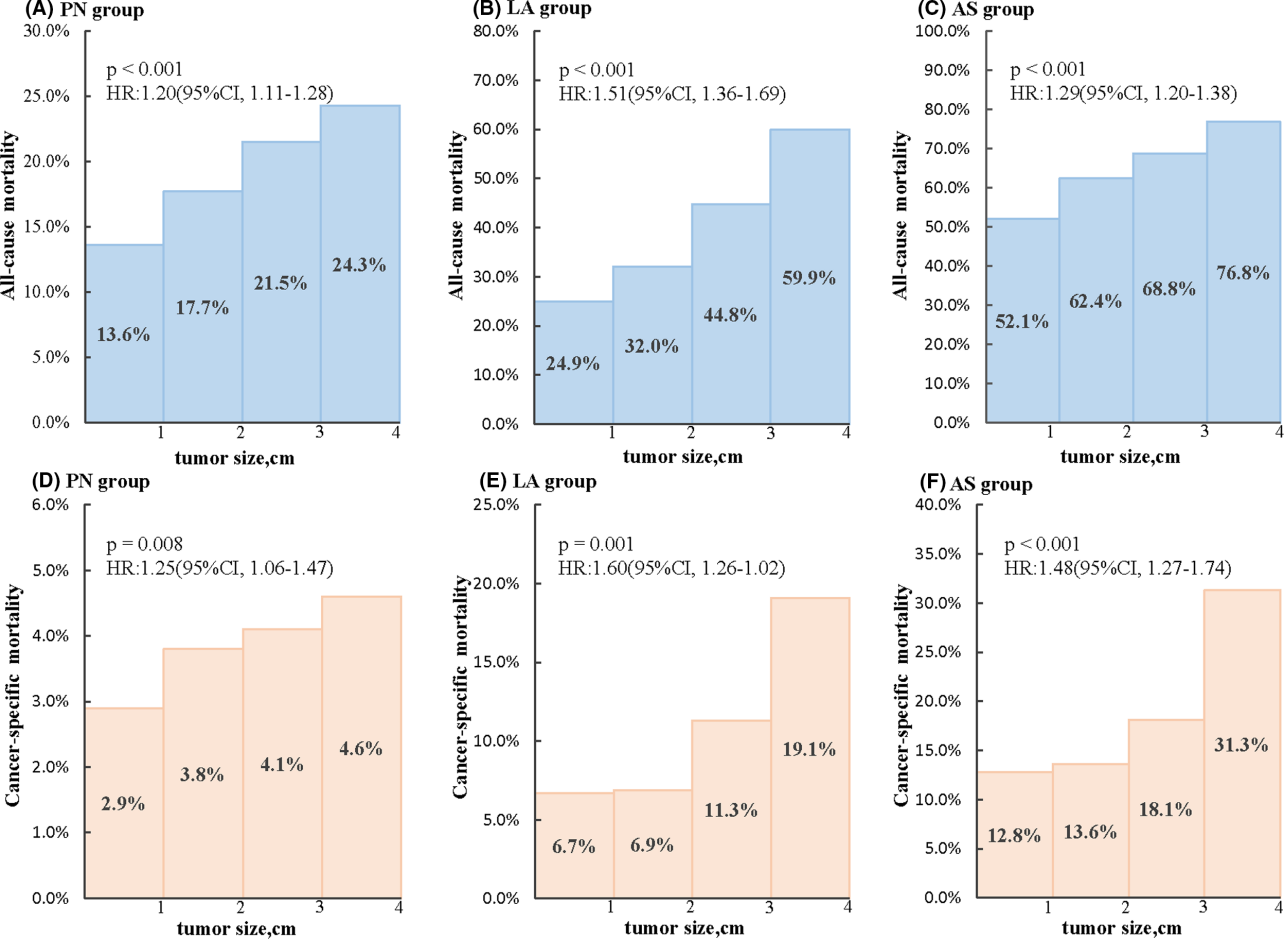
Ten**‐**year mortality associated with tumor size in different treatment groups. Shown are the all‐cause mortality of the PN (A), LA (B), and AS (C) groups and the cancer‐specific mortality of the PN (D), LA (E), and AS (F) groups in different tumor sizes at 10 years. All‐cause mortality and cancer‐specific mortality increase with larger tumor size irrespective of treatment method

To correlate mortality outcomes for treatment with PN, LA, or AS with tumor size, we divided tumor size into four categories: ≤1, >1–2, >2–3, and >3–4 cm in diameter (Figure 2). All‐cause mortality in patients who underwent PN increased with tumor size, from 13.6% in the ≤1 cm group to 24.3% in the >3–4 cm group at 10 years. Higher cancer‐specific mortality also correlated with tumor size in patients who underwent PN, from 2.9% in the ≤1 cm group to 4.6% in the >3–4 cm group at 10 years. Ten‐year all‐cause mortality and cancer‐specific mortality also gradually increased in patients who underwent LA, from 24.9% in the ≤1 cm group to 59.9% in the >3–4 cm group, and from 6.7% in the ≤1 cm group to 19.1% in the >3–4 cm group, respectively. Ten‐year all‐cause mortality associated with AS increased from 52.1% in the ≤1 cm group to 76.8% in >3–4 cm group, and 10‐year cancer‐specific mortality increased from 12.8% in the ≤1 cm group to 31.3% in the >3–4 cm group. Thus, all‐cause mortality and cancer‐specific mortality increased with larger tumor size irrespective of the treatment method.

To determine whether tumor size was an independent risk factor for outcome in T1a RCC, we conducted multivariate Cox regression analyses. As shown in Tables S1 and S2, after adjusting for age, gender, grade, histological subtype, and treatment method, tumor size was an independent prognostic factor of all‐cause mortality and cancer‐specific mortality in T1a RCC (all *p* < 0.001). Therefore, when the patients with T1a RCC have the treatment of PN or LA, tumor size is an independent predictor which smaller tumor be diagnosed and treated indicate better prognosis even in the same T1a stage. When a patient with T1a RCC delay treatment may have risk of larger tumor size and higher mortality even in the same T1a stage.

**FIGURE 3:**
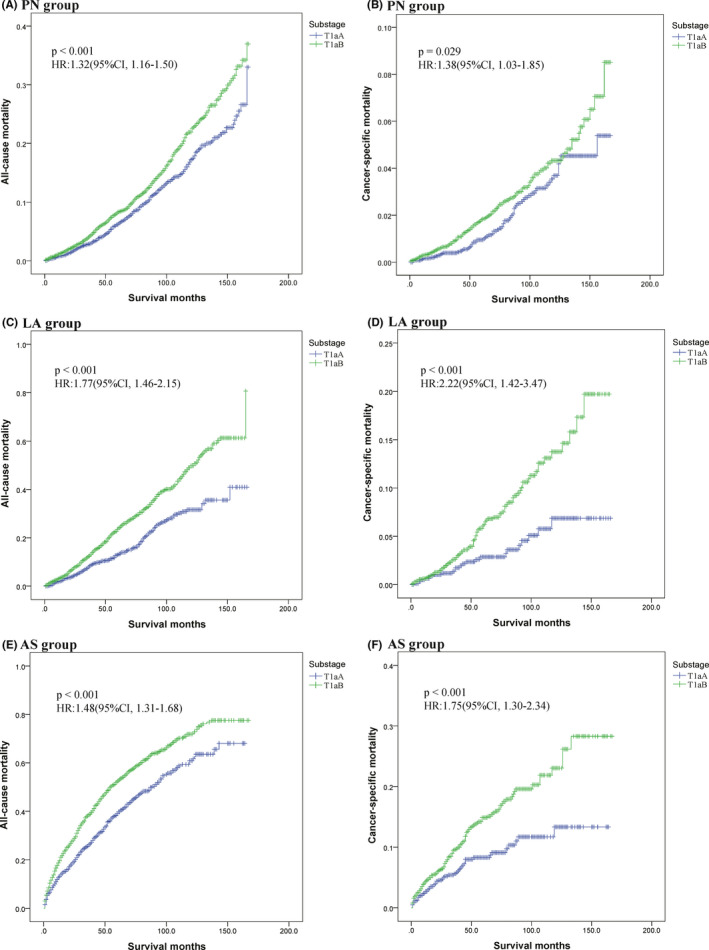
KM curves of all‐cause mortality and cancer‐specific mortality in patients with SRMs in different treatment groups by tumor size (T1aA [≤2 cm] or T1aB [>2–4 cm]). Shown are all‐cause mortality and cancer‐specific mortality curves of patients with T1aA and T1aB RCC as defined by tumor size. (A) KM curves of all‐cause mortality in the PN group. (B) KM curves of cancer‐specific mortality in the PN group. (C) KM curves of all‐cause mortality in the LA group. (D) KM curves of cancer‐specific mortality in the LA group. (E) KM curves of all‐cause mortality in the AS group. (F) KM curves of cancer‐specific mortality in the AS group

Then we further subdivided Stage T1a RCC into T1aA (≤2 cm) and T1aB (>2–4 cm) at the condition of using 2 cm as the cutoff value and conducted KM analyses (Figure 3 **)**. Indeed, in the PN group, all‐cause mortality (HR = 1.32, 95% CI: 1.16–1.50, *p* < 0.001; Figure 3A) and cancer‐specific mortality (HR = 1.38, 95% CI: 1.03–1.85, *p* = 0.029; Figure 3B) were both higher in the T1aB substage compared to the T1aA substage. In addition, all‐cause mortality was significantly higher for patients with T1aB tumors treated with LA compared to patients with T1aA tumors who received the same intervention (HR = 1.77, 95% CI: 1.46–2.15, *p* < 0.001; Figure 3C), and cancer‐specific mortality was similar between these two groups (HR = 2.22, 95% CI: 1.42–3.47, *p* < 0.001; Figure 3D). Similar all‐cause mortality (HR = 1.48, 95% CI: 1.31–1.68, *p* < 0.001; Figure 3E) and cancer‐specific mortality (HR = 1.75, 95% CI: 1.30–2.34, *p* < 0.001; Figure 3F) were also observed among AS group. Therefore, T1aB was associated with significantly higher mortality than T1aA irrespective of treatment method.

To determine whether the new 2 cm threshold of T1a tumors was an independent risk factor for all‐cause mortality and cancer‐specific mortality, we adjusted the tumor substage (T1aA or T1aB) by age, gender, grade, histological subtype, and treatment method in multivariable Cox regression analyses. As shown in Tables 2 and 3, the new substaging system was an independent predictor of all‐cause mortality and cancer‐specific mortality in T1a RCC (all *p* < 0.001). Patients with T1aA RCC in the PN and LA groups had significantly better prognosis than patients with T1aB tumors who received the same therapy. Thus, AS with DI might allow a T1aA tumor to grow to T1aB represents disease progression that is associated with poorer mortality outcomes.

**TABLE 2:** Results of univariate and multivariate Cox regression analyses associated with all‐cause mortality

Variables	Univariate analysis			Multivariate analysis		
	HR	95% CI	*p* value	HR	95% CI	*p* value
Age	4.19	3.80–4.63	**<0.001**	2.79	2.52–3.09	**<0.001**
Gender	1.16	1.08–1.25	**0.001**	1.12	1.03–1.21	**0.005**
Grade	1.42	1.39–1.46	**<0.001**	1.04	1.00–1.07	**0.025**
Histological subtypes	1.35	1.32–1.39	**<0.001**	1.06	1.03–1.10	**0.001**
Treatment	2.70	2.59–2.81	**<0.001**	2.19	2.07–2.31	**<0.001**
Sub‐stage (T1aB vs. T1aA)^a^	1.47	1.35–1.59	**<0.001**	1.40	1.29–1.51	**<0.001**

*Note*: Bold values indicate that the overall data were statistically significant (*p* < 0.05).

Abbreviations: CI, confidence interval; HR, hazard ratio.

^a^ T1aA means tumor size ≤2 cm in diameter; T1aB means tumor size >2 to ≤4 cm in diameter.

**TABLE 3:** Results of univariate and multivariate Cox regression analyses associated with cancer‐specific mortality

Variables	Univariate analysis			Multivariate analysis		
	HR	95%CI	*p* value	HR	95%CI	*p* value
Age	5.97	4.64–7.67	**<0.001**	3.97	3.07–5.13	**<0.001**
Gender	1.18	0.99–1.40	0.065	1.12	0.94–1.33	0.198
Grade	1.46	1.38–1.54	**<0.001**	1.07	1.00–1.15	**0.040**
Histological subtypes	1.33	1.25–1.41	**<0.001**	1.02	0.95–1.09	0.643
Treatment	2.73	2.48–3.00	**<0.001**	2.14	1.90–2.41	**<0.001**
Substage(T1aB vs T1aA)^a^	1.67	1.39–2.02	**<0.001**	1.56	1.30–1.89	**<0.001**

*Note*: Bold values indicate that the overall data were statistically significant (*p* < 0.05).

Abbreviations: CI, confidence interval; HR, hazard ratio.

^a^ T1aA means tumor size ≤2 cm in diameter; T1aB means tumor size >2 to ≤4 cm in diameter.

## 4 DISCUSSION

In this study, we found that patient mortality increased with increasing size of T1a RCC irrespective of treatment type (PN, LA, or AS). Moreover, our data suggest that 2 cm might be a clinically relevant tumor size threshold to classify T1a tumors according to mortality outcomes.

Increasingly, clinicians diagnose RCC at an early stage, owing to improved screening and imaging; thus, performing an adequate risk assessment and assigning patients to therapy and/or follow‐up accordingly is critical to optimize clinical outcomes.^13^, ^14^ PN and LA are the most used surgical options for SRMs, whereas AS is the most used conservative management option.^15^ Currently, AS is established as an acceptable treatment modality with curative intent for patients with SRMs, especially in those whose tumors are smaller than 2 cm in diameter.^16^ We found that the use of AS has increased over time, from 12.4% in 2004 to 20.9% in 2017. A prospective cohort study enrolled 497 patients with a median follow‐up of 2.1 years, in which 274 (55%) patients chose PI and 223 (45%) patients chose AS. The overall survival (OS) rate at 5 years for PI and AS were 92% and 75%, respectively (*p* = 0.06). In that study, the authors found that, in a cohort with up to 5 years of prospective follow‐up, AS was not inferior to PI; however, the mean tumor size of the AS group was significantly smaller than the mean tumor size of the PI group (1.9 cm vs. 2.5 cm; *p* < 0.05).^9^ In another study involving 224 young patients (age ≤ 60 years) with a median follow‐up of 4.9 years, no statistically significant difference was observed in 7‐year OS and cancer‐specific survival between the AS and PI groups; however, the mean tumor size of the AS group was also significantly smaller than the mean tumor size of the PI group (1.5 cm vs. 2.5 cm; *p* < 0.01).^6^ These results might reflect the comfort of patients and urologists with AS as a management strategy, but the value of these findings is probably limited by the inevitable selection bias. In both studies, the mean tumor size in the AS groups was smaller than 2 cm in diameter, and the mean tumor size in the PI groups was larger than 2 cm in diameter. In our study, we found that substage T1aA (≤2 cm) had significantly better prognosis than T1aB (>2–4 cm).

SRMs are not static in terms of the biological characteristics of RCC, and it has been reported that most of the masses will gradually increase over time.^17^ In a meta‐analysis, Chawla et al. showed that RCC grows at a mean rate of 0.09–0.86 cm per year,^11^ and 34/223 (15.2%) patients experienced a growth rate of more than 0.5 cm per year.^9^ Many tumors do not grow for an extended period and then might grow rapidly, which might lead to fast growth of tumors in the AS interstitial phase, resulting in the optimal treatment time being missed.^18^ We found that tumor size is the primary relevant prognostic factor in patients with T1a RCC. The probability of high Fuhrman nuclear grade RCC increases significantly with increase in tumor size.^19^ Another study similarly found that a renal tumor up to 3 cm in diameter, including asymptomatic lesions, showed a significantly higher incidence of high nuclear grade and tumor extension beyond the renal capsule,^20^ which indicates that larger SRMs are highly likely to progress, even though these masses are still considered stage T1a tumors. We found that tumor size was an independent prognostic factor of all‐cause mortality and cancer‐specific mortality in T1a RCC and that delaying treatment of patients with T1a RCC might incur a risk of larger tumor size and higher mortality.

In our large‐sample dataset, we found that all‐cause mortality and cancer‐specific mortality for >2–4 cm T1a (substage T1aB) tumors were higher than those for ≤2 cm T1a (substage T1aA) tumors in the PN, LA, and AS groups. Thus, we introduce a new substaging paradigm as an independent predictor of clinical outcome in T1a RCC, where development of T1aA tumors to T1aB tumors indicates progression to disease with poorer clinical outcomes. For example, for a patient with a 1.8‐cm diameter renal mass (T1aA), the tumor might increase to 2.3 cm (T1aB) under AS after 1 year. Although the tumor was also classified as stage T1a RCC, the actual prognosis has worsened. According to our results, if the patient chose PN or LA 1 year late, the increase of 10‐year all‐cause mortality and 10‐year cancer‐specific mortality would be 30.4% and 22.9% (PN group), 55.1% and 100% (LA group), respectively. During AS of patients with SRMs, the tumor growth rate of more than 15% of patients was higher than 0.5 cm per year,^9^ and currently, there is no accurate predictor for tumor growth rate, AS for SRMs patients has risk of tumor progression.

Another important reason that AS for patients with SRMs has been accepted is that over‐diagnosis and over‐treatment have been recognized in a variety of accidentally detected cancers.^21^ Several surgical series have shown that 20%–25% of SRMs are diagnosed as benign on postoperative pathology,^22^, ^23^, ^24^ and it is desirable to avoid the over‐treatment of benign lesions.^25^ In a review of 57 studies that recruited a total of 5228 patients, the overall median diagnostic rate of renal tumor biopsy was 92%.^26^ Another study showed that the accuracy of image‐guided tumor biopsy can reach 95% for SRMs with a tumor size smaller than 2 cm in diameter.^27^ Therefore, performing a biopsy before a treatment decision is made can help accurately distinguish between benign and malignant SRMs, especially for tumors smaller than 2 cm.

Our study is limited by its retrospective nature and is, thus, prone to an inevitable selection bias, which is common in all non‐prospective, non‐randomized studies. However, we are still confident that our findings, based on a large patient population, are useful for making clinical decisions about SRMs. We assert that 2 cm should be a new breakpoint to assign a substage associated with the prognosis of T1a RCC.

## 5 CONCLUSION

We found tumor size to be an independent risk factor of prognosis of T1a RCC and that patients with tumors >2–4 cm in diameter (substage T1aB) had significantly worse survival than patients with tumors ≤2 cm in diameter (substage T1aA). We believed that this new size threshold of 2 cm might accurately predict prognosis and indicate tumor progression, which would facilitate the clinicians in choosing the appropriate therapeutic treatment and management of the patients. AS as a treatment option for patients with SRMs should be chosen with caution, because no clinical variables or biomarkers have been found to accurately predict the rate of tumor growth or the patient population at risk of progression during AS.

## CONFLICT OF INTEREST

The authors declare that this research was conducted in the absence of any commercial or financial relationships that could be construed as a potential conflict of interest.

## AUTHOR CONTRIBUTIONS

Junhang Luo, Yi Hou, and Wei Chen: Project development. Mukhtar A. Mumin, Junhang Luo, Yi Hou, Hongde Song, Haishan Lin, Lei Tan, Chengpeng Gui, Mingxiao Zhang, and Liangmin Fu: Data collection or management. Yiming Tang, Pengju Li, Mukhtar A. Mumin, Junhang Luo, Yi Hou, Hongde Song, and Haishan Lin: Data analysis. Yiming Tang, Fei Liu, Xiaopeng Mao, and Pengju Li: Manuscript writing. Yiming Tang, Lei Tan, Chengpeng Gui, Mingxiao Zhang, and Liangmin Fu: Statistical analysis.

## ETHICS APPROVAL

The data of this research were obtained from the public database and no ethical approval was required.

## Supporting information


**SUPPLEMENTARY TABLE 1** Results of univariate and multivariate Cox regression analyses associated with all‐cause mortality
**SUPPLEMENTARY Table 2** Results of univariate and multivariate Cox regression analyses associated with cancer‐specific mortality
**SUPPLEMENTARY Figure 1** Proportion of treatment with the year at diagnosis in patients with SRMs (tumor size ≤2 cm in diameter). Stacked bar chart shows that the proportion of AS was higher for tumors smaller than 2 cm in diameter, increasing from 11.4% in 2004 to 24.2% in 2017.
**SUPPLEMENTARY Figure 2** Proportion of treatment with the year at diagnosis in patients with SRMs (tumor size >2–4 cm in diameter). Stacked bar chart shows that the proportion of AS increased with the year of diagnosis in tumors >2–4 cm in diameter, from 13.1% in 2004 to 19.4% in 2017.

## Data Availability

The data that support the findings of the current study are available from the public Surveillance, Epidemiology, and End Results datebase (https://seer.cancer.gov/).
