# Supplementary material for: The impact of tumor size on the survival of patients with small renal masses: A population–based study
Source: Cancer Med. 2022 Mar 1;11(12):2377–85. doi: 10.1002/cam4.4595 (PMC9189465; doi:10.1002/cam4.4595)
Supplement: Supplementary file 1 — SUPPLEMENTARY TABLE 1 Results of univariate and multivariate Cox regression analyses associated with all‐cause mortality SUPPLEMENTARY Table 2 Results of univariate and multivariate Cox regression analyses associated with cancer‐specific mortality SUPPLEMENTARY Figure 1 Proportion of treatment with the year at diagnosis in patients with SRMs (tumor size ≤2 cm in diameter). Stacked bar chart shows that the proportion of AS was higher for tumors smaller than 2 cm in diameter, increasing from 11.4% in 2004 to 24.2% in 2017. SUPPLEMENTARY Figure 2 Proportion of treatment with the year at diagnosis in patients with SRMs (tumor size >2–4 cm in diameter). Stacked bar chart shows that the proportion of AS increased with the year of diagnosis in tumors >2–4 cm in diameter, from 13.1% in 2004 to 19.4% in 2017. [file CAM4-11-2377-s001.docx]

**SUPPLEMENTARY TABLE 1** Results of univariate and multivariate Cox regression analyses associated with all–cause mortality

| Variables | Univariate analysis | | | Multivariate analysis | | | |
| --- | --- | --- | --- | --- | --- | --- | --- |
|  | HR | 95% CI | *p* value |  | HR | 95% CI | *p* value |
| Age | 4.19 | 3.80–4.63 | **<0.001** |  | 2.78 | 2.51–3.08 | **<0.001** |
| Gender | 1.16 | 1.08–1.25 | **0.001** |  | 1.11 | 1.02–1.19 | **0.011** |
| Grade | 1.42 | 1.39–1.46 | **<0.001** |  | 1.03 | 1.00–1.07 | **0.026** |
| Histological subtypes | 1.35 | 1.32–1.39 | **<0.001** |  | 1.06 | 1.03–1.10 | **<0.001** |
| Treatment | 2.70 | 2.59–2.81 | **<0.001** |  | 2.20 | 2.08–2.32 | **<0.001** |
| Tumor size | 1.30 | 1.24–1.36 | **<0.001** |  | 1.28 | 1.23–1.34 | **<0.001** |

Bold values indicate that the overall data were statistically significant (*p* < 0.05).

HR, hazard ratio; CI, confidence interval.

**SUPPLEMENTARY TABLE 2** Results of univariate and multivariate Cox regression analyses associated with cancer–specific mortality

| Variables | Univariate analysis | | | Multivariate analysis | | | |
| --- | --- | --- | --- | --- | --- | --- | --- |
|  | HR | 95%CI | *p* value |  | HR | 95%CI | *p* value |
| Age | 5.97 | 4.64–7.67 | **<0.001** |  | 3.95 | 3.06–5.10 | **<0.001** |
| Gender | 1.18 | 0.99–1.40 | 0.065 |  | 1.10 | 0.93–1.31 | 0.276 |
| Grade | 1.46 | 1.38–1.54 | **<0.001** |  | 1.07 | 1.00–1.15 | **0.043** |
| Histological subtypes | 1.33 | 1.25–1.41 | **<0.001** |  | 1.02 | 0.95–1.10 | 0.560 |
| Treatment | 2.73 | 2.48–3.00 | **<0.001** |  | 2.15 | 1.90–2.42 | **<0.001** |
| Tumor size | 1.45 | 1.31–1.61 | **<0.001** |  | 1.43 | 1.29–1.58 | **<0.001** |

Bold values indicate that the overall data were statistically significant (*p* < 0.05).

**
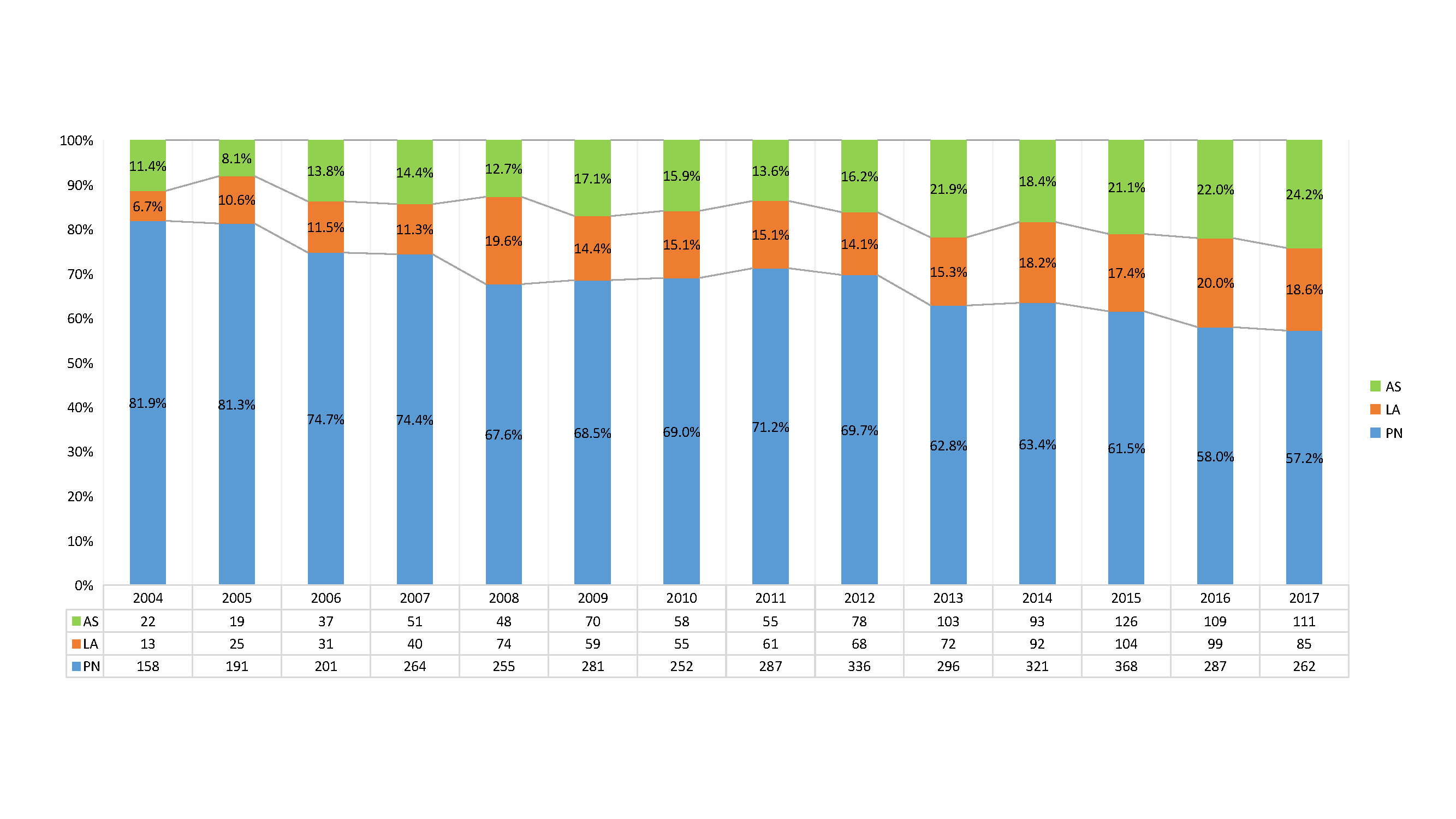
**

**SUPPLEMENTARY FIGURE 1** Proportion of treatment with the year at diagnosis in patients with SRMs (tumor size ≤2 cm in diameter). Stacked bar chart shows that the proportion of AS was higher for tumors smaller than 2 cm in diameter, increasing from 11.4% in 2004 to 24.2% in 2017.


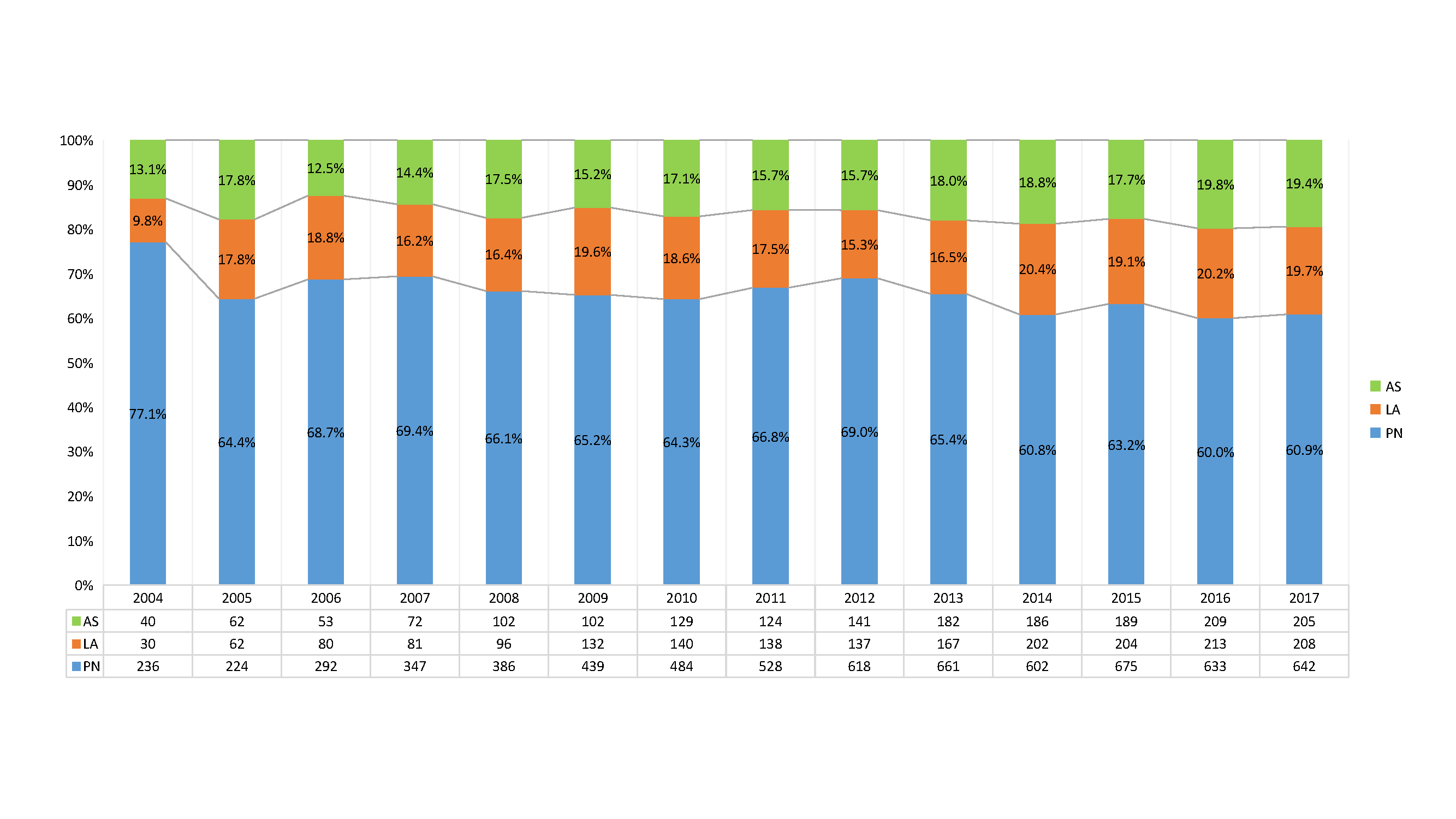


**SUPPLEMENTARY FIGURE 2** Proportion of treatment with the year at diagnosis in patients with SRMs (tumor size >2–4 cm in diameter). Stacked bar chart shows that the proportion of AS increased with the year of diagnosis in tumors >2–4 cm in diameter, from 13.1% in 2004 to 19.4% in 2017.
